# Catheter-Associated *Trichosporon japonicum* Fungemia in a Patient with Diffuse Large B-Cell Lymphoma Following CAR-T Cell Therapy: A Case Report and Literature Review

**DOI:** 10.3390/jof12050320

**Published:** 2026-04-27

**Authors:** Liyan Mao, Shaozhen Yan, Lei Tian, Cui Jian, Yue Wang, Ziyong Sun, Zhongju Chen

**Affiliations:** Department of Laboratory Medicine, Tongji Hospital, Tongji Medical College, Huazhong University of Science and Technology, Wuhan 430030, China; liyanmao@tjh.tjmu.edu.cn (L.M.); ysz209@163.com (S.Y.); iso15189@126.com (L.T.); jiancui_tj@126.com (C.J.); wangyue@tjh.tjmu.edu.cn (Y.W.); zysun@tjh.tjmu.edu.cn (Z.S.)

**Keywords:** *Trichosporon japonicum*, CAR-T cell therapy, catheter-related fungemia, voriconazole, amphotericin B

## Abstract

Background: *Trichosporon japonicum* is a rare but highly lethal pathogen causing fungemia in immunocompromised patients. With the expanding use of chimeric antigen receptor T (CAR-T) cell therapy, the spectrum of opportunistic fungal infections is changing, yet data on *T. japonicum* infections in this setting remain scarce. Case Presentation: A 69-year-old man with diffuse large B-cell lymphoma developed catheter-associated fungemia after CAR-T cell reinfusion. He initially presented with neck pain and white oral mucosal patches, followed by fever four days later. *T. japonicum* was isolated from both peripheral blood and central venous catheter tip cultures, identified by microscopic examination, mass spectrometry, and molecular sequencing. Antifungal prophylaxis was initiated before fever onset based on close monitoring of white blood cell count, procalcitonin, interleukin-6, and C-reactive protein; treatment was subsequently adjusted according to species identification and antifungal susceptibility results. Infection was controlled within two weeks after catheter removal and immune recovery. The patient remained well at six-month follow-up. Conclusion: This case adds to the limited literature on *T. japonicum* fungemia in patients receiving CAR-T therapy. Our experience, together with a review of the literature, underscores that successful management requires prompt catheter removal, immune restoration, and combination therapy with voriconazole and amphotericin B, as echinocandin monotherapy should be avoided. Awareness of this pathogen in immunocompromised patients is critical.

## 1. Introduction

*Trichosporon* species are emerging opportunistic yeasts widely distributed in nature, inhabiting soil, decomposed wood, water, and various animals [1]. They also colonize human skin, gastrointestinal tract, and respiratory tract as part of the normal flora. Invasive trichosporonosis has been increasingly reported over the past decades, particularly in immunocompromised patients [2,3]. Among *Trichosporon* species, *Trichosporon asahii* is the most common pathogen causing invasive infections, with fungemia being the predominant manifestation, with mortality rates ranging from 30% to 90% [4,5]. However, data on non-asahii *Trichosporon* species remain limited due to their low isolation rates and difficulties in accurate identification. Conventional laboratory methods often fail to differentiate *Trichosporon* species reliably, potentially leading to misidentification and suboptimal clinical management [6].

*Trichosporon japonicum* is an exceptionally rare pathogen, with only a few cases of invasive infection documented in the literature. Available evidence suggests that *T. japonicum* fungemia carries a poor prognosis, particularly in patients with hematologic malignancies, and reports of successful treatment with long-term survival are scarce [7,8]. Moreover, no standardized antifungal regimens exist for this rare species, and susceptibility patterns vary considerably among different strains. The increasing use of novel immunotherapies, such as chimeric antigen receptor T (CAR-T) cell therapy, further expands the population at risk for unusual opportunistic infections, yet data on *T. japonicum* infections in this context are virtually absent.

Herein, we report a case of catheter-associated *T. japonicum* fungemia in a patient with diffuse large B-cell lymphoma (DLBCL) who underwent CAR-T cell therapy. The infection was successfully managed with timely catheter removal, immune recovery, and susceptibility-guided antifungal therapy. We also review the literature on *T. japonicum* infections to improve recognition and management of this rare but life-threatening pathogen.

## 2. Case Presentation

A 69-year-old man was admitted to our hospital in March 2021 with a diagnosis of DLBCL. He subsequently received four cycles of RM-CHOP (rituximab, cyclophosphamide, doxorubicin, vincristine, and high-dose methotrexate), followed by four cycles of R-Gemox (rituximab, gemcitabine, and oxaliplatin) combined with oral ibrutinib as salvage chemotherapy. In November 2021, the patient underwent chimeric antigen receptor T (CAR-T) cell therapy. He had no history of diabetes mellitus but developed hyperglycemia during hospitalization. He also had a history of prior exposure to multiple antimicrobial agents.

Approximately one month after CAR-T cell infusion, the patient developed neck pain that worsened with swallowing. Physical examination revealed white mucosal patches in the oral cavity; however, smear of the oral lesions was negative. Laboratory findings at that time are summarized in Table 1. Markedly elevated inflammatory markers were observed, including interleukin-6 (IL-6, 3116.00 pg/mL) and C-reactive protein (CRP, 44.50 mg/L), while procalcitonin (PCT) was 0.13 ng/mL. In contrast, white blood cell count (WBC, 1.24 × 10^9^/L) and absolute neutrophil count (0.04 × 10^9^/L) were profoundly decreased. Bone marrow examination confirmed severe bone marrow suppression. The patient also exhibited persistent hyperglycemia during this period.

Intravenous micafungin (150 mg once daily) was initiated as antifungal prophylaxis. Despite this, inflammatory markers continued to rise, with CRP increasing to 88.2 mg/L, PCT to 0.24 ng/mL, and IL-6 exceeding 5000 pg/mL. Antifungal therapy was then switched to amphotericin B (2 mg/kg once daily). Although IL-6 showed a transient decrease, it subsequently rebounded. Three days later, the patient developed intermittent high fever. Blood cultures were obtained from both the central venous catheter and a peripheral vein to identify potential pathogens.

**Figure 1:**
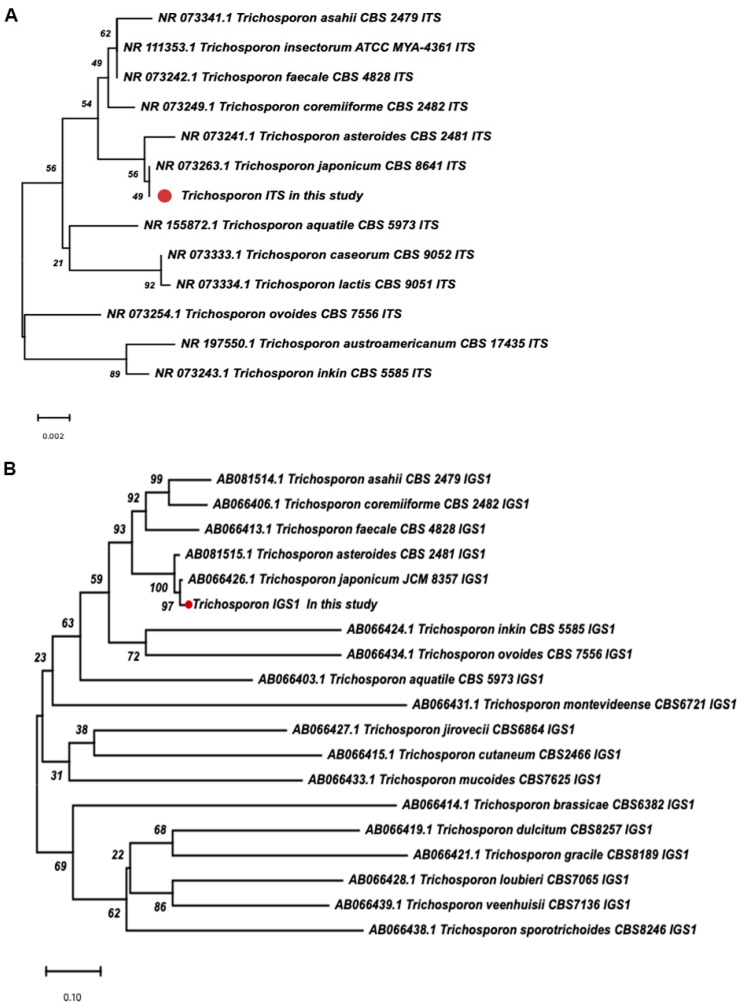
Phylogenetic tree of *Trichosporon japonicum* based on ITS (**A**) and IGS1 (**B**) gene sequences. The trees were constructed using the Neighbor-Joining method in MEGA 11 software. Bootstrap values (based on 1000 replicates) are shown at the branch nodes. The scale bar indicates the genetic distance per nucleotide site. Red bullets indicate the sequence from the present study.

The following day, the peripheral blood culture turned positive, and microscopic examination revealed arthroconidia suggestive of *Trichosporon* species. At this point, the amphotericin B dose was increased to 4 mg/kg once daily, and voriconazole (4 mg/kg twice daily) was added. The isolate was identified as *T. japonicum* by matrix-assisted laser desorption/ionization time-of-flight mass spectrometry (MALDI-TOF MS, Bruker Biotyper, Bremen, Germany) with a high confidence score of 2.19. In parallel, VITEK 2 compact identified the isolate as *T. asahii* (97% probability). Definitive identification was achieved by internal transcribed spacer (ITS) sequencing (sequences have been deposited in the GenBank database under accession numbers PZ161416) and further confirmed by intergenic spacer 1 (IGS1) sequencing (accession numbers PZ302131). Phylogenetic trees were constructed based on ITS (Figure 1A) and IGS1 (Figure 1B) sequences (original sequence data were shown in Supplementary File S1), confirming the pathogen as *T. japonicum*.

Antifungal susceptibility testing was performed following Clinical and Laboratory Standards Institute (CLSI) guidelines. The minimum inhibitory concentrations (MICs) for this isolate are shown in Table 2: anidulafungin (AND, 8 µg/mL), caspofungin (CAS, 8 µg/mL), micafungin (MF, 8 µg/mL), amphotericin B (AMB, 1.0 µg/mL), fluconazole (FZ, 256 µg/mL), itraconazole (IZ, 16 µg/mL), posaconazole (PZ, 4 µg/mL), 5-fluorocytosine (5-FC, 64 µg/mL), and voriconazole (VOR, 8 µg/mL). Serum cryptococcal antigen testing (CrAg^®^ LFA kit, IMMY, Norman, OK, USA) was positive at a titer of 1:10. The central venous catheter was subsequently removed, and catheter tip culture also yielded *T. japonicum* with an identical antifungal susceptibility profile to the peripheral blood isolate.

The combination of amphotericin B and voriconazole was continued for 16 days. Following initiation of this regimen, IL-6 levels decreased rapidly and continuously, followed by gradual declines in PCT and CRP. The patient’s febrile symptoms resolved. Leukocyte and neutrophil counts gradually recovered with granulocyte colony-stimulating factor (G-CSF) support. Follow-up blood cultures obtained on day 6 and day 14 of antifungal therapy were both negative. The infection was effectively controlled, and the patient resumed treatment for his underlying hematologic malignancy. He was discharged two months later and remained in good condition throughout six months of subsequent follow-up.

## 3. Discussion

*T. japonicum* is an extremely rare opportunistic fungus associated with invasive infections and high mortality [3]. Only sporadic cases have been reported in the literature, with most patients having poor outcomes. We present a case of catheter-associated *T. japonicum* fungemia in a patient with DLBCL who underwent CAR-T cell therapy and was successfully treated. This report provides new data on the clinical characteristics and management of rare fungal infections in the context of emerging immunotherapies.

This case highlights the challenge of accurate identification of *T. japonicum*. VITEK 2 compact misidentified the isolate as *T. asahii* with 97% confidence, whereas MALDI-TOF MS correctly identified it as *T. japonicum* with a score of 2.19. However, MALDI-TOF MS is database-dependent and may not distinguish closely related species; for example, commercial databases cannot differentiate *T. japonicum* from *Trichosporon asteroides* [17]. Thus, while MALDI-TOF provided a correct genus-level match, it is not reliable for definitive species identification. Molecular methods offer higher resolution. ITS sequencing initially identified *T. japonicum*, and the ITS-based tree (Figure 1A) placed it close to *T. japonicum* reference strains. However, ITS has limited discriminatory power for some *Trichosporon* species, and the moderate bootstrap support in Figure 1A suggests it may not fully resolve *T. japonicum* from *T. asteroides*. We therefore performed IGS1 sequencing, the preferred marker for *Trichosporon* species delineation. The IGS1 sequence showed 97.89% identity to the *T. japonicum* type strain (JCM 8357) and 95.60% identity to the *T. asteroides* type strain (CBS 2481). Interestingly, BLASTn (http://blast.ncbi.nlm.nih.gov/Blast.cgi, accessed 19 April 2026) ranked multiple *T. asteroides* clinical isolates above the *T. japonicum* type strain, primarily due to higher query coverage (96% vs. 75%). This reflects a known caveat: BLAST (http://blast.ncbi.nlm.nih.gov/Blast.cgi, accessed 19 April 2026) prioritizes local alignment length and coverage over overall evolutionary relatedness. Therefore, BLAST results should be interpreted with caution for closely related species. The gold standard is phylogenetic analysis based on type strains. As shown in Figure 1B, our IGS1 sequence clustered with the *T. japonicum* type strain (JCM 8357) with strong bootstrap support (98%), clearly separated from *T. asteroides* (CBS 2481). Based on this evidence, the isolate was definitively identified as *T. japonicum*. In summary, conventional biochemical methods are reliable only at the genus level [7,17]. MALDI-TOF is rapid but database-dependent; ITS has limited resolution. For definitive species-level identification of *Trichosporon*, IGS1 sequencing with phylogenetic analysis using type strains is strongly recommended, especially in immunocompromised patients with suspected rare fungal infections.

Another noteworthy finding was a positive serum cryptococcal antigen test (titer 1:10). Certain *Trichosporon* species produce polysaccharides immunologically similar to *Cryptococcus neoformans* capsular glucuronoxylomannan, leading to cross-reactivity [18]. A similar phenomenon was reported in a heart transplant recipient with *T. japonicum* pericardial effusion who tested positive for cryptococcal antigen (titer 1:80) [8]. Strains from disseminated infections produce more glucuronoxylomannan-like polysaccharides than those causing superficial infections, which may explain this observation [18]. Clinicians should be aware that a positive cryptococcal antigen test does not equate to cryptococcal infection and requires correlation with culture and molecular results.

*T. japonicum* was first isolated from air and named by Sugita and Nakase in 1998 [19], with the first clinical case reported in 2008 [7]. We systematically reviewed reported cases of invasive *T. japonicum* infection (Table 3). Since 2008, including the present case, eight cases have been reported (five males, three females; age range 8–50 years). Underlying conditions included hematologic malignancies and solid organ transplantation. Infection types included fungemia (2 cases) [8,12], urinary tract infection (2 cases) [14], respiratory infection (1 case) [7], pericardial effusion (1 case) [16], and cutaneous infection (2 cases, non-invasive) [10]. Regarding diagnostic methods, MALDI-TOF MS was used in five cases; IGS1 sequencing was performed in three cases, including this case; two cases employed ITS plus 28S D1/D2 sequencing; and two cases used ITS sequencing alone or other molecular typing methods. Most invasive cases were associated with indwelling catheters: both fungemia patients had central venous catheters, both urinary tract infection patients had urinary catheters, and the pericardial effusion patient had a drainage tube. The overall mortality rate for invasive infections was 60% (4/6). Surviving cases included a patient with post-myocarditis fungemia [12], a kidney transplant recipient with urinary tract infection [14], and the present case—the first successfully treated hematologic malignancy patient after CAR-T therapy.

Catheter-related infection is the predominant form of invasive *T. japonicum* infection. In the present case and that reported by Bongomin et al. [12], the pathogen was cultured from both peripheral blood and catheter tip, and infection was controlled after catheter removal along with antifungal treatment. Albitar-Nehme et al. [8] reported an 8-year-old child with acute lymphoblastic leukemia who achieved blood culture clearance with amphotericin B and voriconazole despite retaining the central venous catheter, though the patient ultimately died from underlying disease progression. In urinary tract infection cases reported by Li et al. [14], both kidney transplant recipients had urinary catheters; the patient who underwent catheter removal plus antifungal therapy achieved infection control and survived, while the patient without catheter removal had persistently positive urine cultures after 15 days of antifungal therapy and eventually died. European Confederation of Medical Mycology/International Society for Human and Animal Mycology (ECMM/ISHAM) global guidelines recommend considering central venous catheter removal in patients with *Trichosporon* bloodstream infection [2], as *Trichosporon* readily forms biofilms on biomedical materials [20]. In clinical practice, the necessity of catheter removal should be actively evaluated in high-risk patients with indwelling devices, alongside timely and appropriate antifungal treatment and immune support.

Host immune status is another critical determinant of outcome. Over 80% of trichosporonosis cases occur in patients with hematologic malignancies who have persistent neutropenia, neutrophil dysfunction, or prior echinocandin exposure [3,20]. The present case clearly illustrates this: the patient had severe neutropenia (0.04 × 10^9^/L) at infection onset, and infection progressed despite antifungal therapy; with G-CSF support, neutrophil recovery was accompanied by rapid decline in IL-6 and eventual infection control. Immune suppression after CAR-T therapy is more complex than conventional chemotherapy, involving sustained B-cell deficiency and hypogammaglobulinemia. Antifungal therapy and immune support (G-CSF) should be pursued in parallel to improve outcomes.

In the present case, the addition of voriconazole to amphotericin B was prompted by the microscopic finding of arthroconidia in the positive blood culture on Day 6 (Table 1). The combination was started on Day 7, before antifungal susceptibility results were available (reported on Day 8). This early morphology-driven intervention, together with catheter removal and neutrophil recovery, was critical for achieving the successful control of infection. Notably, the patient had been receiving micafungin prophylaxis before fungemia developed, indicating breakthrough infection despite echinocandin coverage. The failure of echinocandin prophylaxis is consistent with the intrinsic resistance of basidiomycetous yeasts, including *Trichosporon* species. Susceptibility testing later confirmed high MICs to echinocandins (Table 2), validating the clinical observation. Due to the paucity of *T. japonicum* clinical isolates, no species-specific breakpoints exist, and current studies reference CLSI or EUCAST criteria for *Candida* or *Cryptococcus*. We systematically reviewed susceptibility data [7,8,9,10,11,12,13,14,15,16] from reported *T. japonicum* isolates (Table 2), revealing the following patterns: (1) universal resistance or poor susceptibility to echinocandins (MICs often >8 µg/mL), including the present isolate. This aligns with the intrinsic resistance of basidiomycetes and explains why micafungin prophylaxis failed in our patient, underscoring that echinocandins should be avoided when *Trichosporon* infection is suspected. (2) Significant inter-strain variability in triazole susceptibility, with voriconazole MICs ranging from 0.015 to >8 µg/mL; the present isolate had a voriconazole MIC of 8 µg/mL, similar to isolates from China [14] and France [16]. (3) Relatively stable susceptibility to amphotericin B (MIC range 0.25–4 µg/mL), with the present isolate at 1.0 µg/mL. (4) Universally high MICs to fluconazole and flucytosine. Different susceptibility testing methods may yield discordant results [14], highlighting the need to interpret MICs in conjunction with clinical response. Despite a high voriconazole MIC, the present isolate was successfully treated with amphotericin B and voriconazole combination, possibly due to drug synergy, coverage of resistant subpopulations, or reduced reliance on drug activity alone after catheter removal and neutrophil recovery. All successfully treated invasive cases (present case, Bongomin et al. [12], and the surviving urinary tract infection case from Li et al. [14]) received voriconazole-containing combination regimens, consistent with ECMM/ISHAM guideline recommendations [2].

This study has several limitations. As a single case report, the generalizability of our findings is limited. Long-term follow-up data are incomplete, as the patient was lost to follow-up after July 2022. The absence of species-specific antifungal breakpoints for *Trichosporon* limits interpretation of susceptibility results. Finally, retrospective analysis is subject to inherent information bias.

In conclusion, successful management of *T. japonicum* infection is multifactorial. *T. japonicum* is a rare but highly lethal opportunistic fungus primarily affecting immunocompromised patients. Based on this case and literature review, we offer the following clinical recommendations: (1) maintain a high index of suspicion in at-risk populations, including patients receiving CAR-T therapy, those with neutropenia, and those with indwelling catheters; (2) use MALDI-TOF MS as a rapid screening tool while recognizing its limitations in distinguishing closely related species, and confirm species identification by IGS1 sequencing as the gold standard for *Trichosporon*; (3) initiate antifungal therapy with voriconazole combined with amphotericin B, and avoid echinocandin monotherapy; (4) actively evaluate catheter removal and provide immune support with G-CSF; (5) monitor inflammatory markers such as IL-6 and CRP dynamically to guide early detection and treatment adjustment. An international multicenter case registry is needed to accumulate more data and facilitate the development of evidence-based guidelines for this rare but devastating infection.

## Figures and Tables

**Table 1:** Timeline of *Trichosporon japonicum* catheter-associated fungemia course.

Timeline	WBC (×10^9^/L)	PCT (ng/mL)	IL-6 (pg/mL)	CRP (mg/L)	Clinical Features and Investigations	Treatment
Nine months of hospital stay	/	/	/	/	diffuse large B-cell lymphoma	Bridging chemotherapy CAR T-cells therapy Infusions, multiple antibiotic prophylaxis
Day 1	1.24	/	3116.00	/	Neck pain, leukoplakia of the oral mucosa	MF (150 mg intravenously once a day)
Day 2	0.50	0.13	5000.00	44.50	Same as above	MF
Day 3	1.43	0.24	426.70	88.20	/	AMB (2 mg/kg intravenously once a day)
Day 4	1.36	0.21	927.70	/	Blood culture (Central venous catheter blood and venous blood)	AMB
Day 5	0.44	/	2202.00	/	Fever	AMB
Day 6	/	/	/	/	Venous blood culture positive, and gram staining showed arthroconidia	AMB
Day 7	0.34	1.53	387.10	88.20	/	AMB (4 mg/kg intravenously once a day) + VOR (4 mg/kg intravenously twice a day)
Day 8	0.53	0.85	349.40	64.20	Identification of *T. japonicum* on MALDI-TOF MS, report the results of fungal susceptibility	AMB + VOR
Day 9	0.91	0.51	235.70	/	Remove the catheter and culture	AMB + VOR
Day 10	1.05	0.54	142.60	/	Two sets of blood cultures	AMB + VOR
Day 11	1.19	0.43	73.13	151.60	Catheter culture positive, Identification of *T. japonicum* on MALDI-TOF MS	AMB + VOR
Day 17	/	/	/	/	Two sets of blood cultures	AMB + VOR
Day 22	/	/	/	/	Four sets of blood cultures all negative	AMB + VOR
Two months later	/	/	/	/	Discharge	/

Note: / denotes missing data or omitted description; WBC: white blood cell; PCT: procaicltonin; IL-6: interleukin 6; CRP, C-reactive protein; MALDI-TOF MS, matrix-assisted laser desorption/ionization-time of flight mass spectrometry; CAR, chimeric antigen receptor; MF: micafungin; AMB: amphotericin B; VOR: voriconazole.

**Table 2:** Minimal Inhibitory Concentrations of Multiple Antifungal Agents Against previous reported clinical isolates of *Trichosporon japonicum*.

Author, Published Year	Identification Method	AST Method	Region	Strains Tested	MIC, mg/L								
					AMB	5-FC	FZ	MF	AND	IZ	PZ	CAS	VOR
Rodriguez-Tudela, J.L. et al., 2005 [9]	IGS1 sequencing	EUCAST/CLSI	Chile	2	0.25–1.0	4–32	0.12–1.0	N.A.	N.A.	0.5	N.A.	N.A.	0.015–0.03
Ağirbasli, H et al., 2008 [7]	IGS1 sequencing	E-test	Japan	1	1	N.A.	0.032	N.A.	N.A.	0.75	N.A.	N.A.	0.032
Taj-Aldeen, S.J. et al., 2009 [10]	ITS + 28S D1/D2 sequencing	CLSI	Netherlands	2	4–16	N.A.	4	N.A.	N.A.	0.125	N.A.	N.A.	0.063–0.125
Kalkanci, A. et al., 2010 [11]	IGS1 sequencing	ASTY colorimetric	Turkey	1	1	8	2	0.5	N.A.	0.5	N.A.	N.A.	0.06
Bongomin, F. et al., 2019 [12]	ITS + 28S D1/D2 sequencing; MALDI-TOF MS	EUCAST	UK	1	1	8	1	>4	N.A.	0.125	N.A.	N.A.	0.06
do Espirito Santo, E.P.T. et al. 2020 [13]	IGS1 sequencing	CLSI	Brazil	1	4	N.A.	2	N.A.	N.A.	1	N.A.	N.A.	N.A.
Li, T. et al., 2020 [14]	ITS sequencing; MALDI-TOF MS	ATB, E-test	China	2	0.5/0.5 0.094/0.19	>16/>16 N.A.	>128/>128 >256/>256	N.A.	N.A.	>4/>4 0.38/0.75	N.A.	N.A. >32/>32	>8/>8 2/2
Ahangarkani, F. et al., 2021 [15]	MALDI-TOF MS	CLSI	Iran	4	0.5–2	4–64	0.5–64	4–8	8	0.16–2	0.016–0.5	N.A.	0.016–2
Albitar-Nehme, S. et al., 2022 [8]	MALDI-TOF MS; Molecular Typing ^a^	CLSI	Italy	1	0.25	N.A.	1	>8	>8	0.12	0.12	8	0.03
Menu, E. et al., 2022 [16]	IGS1 sequencing; MALDI-TOF MS	Yeast-One, E-test	France	1	0.5	4	4	>8	>8	0.12	0.25/0.75	>8	0.12/0.125
This study	ITS + IGS1 sequencing; MALDI-TOF	CLSI	China	1	1	64	256	8	8	16	4	8	8

Note: MIC: Minimal Inhibitory Concentration; AST: Antifungal susceptibility testing; AMB: amphotericin B; 5-FC:5-fluorocytosine; FZ: fluconazole; MF: micafungin; AND: anidulafungin; IZ: itraconazole; PZ: posaconazole; CAS: caspofungin; VOR: voriconazole; EUCAST, European Committee on Antimicrobial Susceptibility Testing; CLSI, Clinical & Laboratory Standards Institute; ATB, ATB Fungus 3 system. N.A. denotes Not available. ^a^ Albitar-Nehme et al. reported using the MicroSEQ™ 500 16S rDNA PCR Kit (Thermo Fisher Scientific, Waltham, MA, USA) for molecular typing, a method primarily designed for bacterial identification.

**Table 3:** Summary of previous reported cases of *Trichosporon japonicum*.

Author	Sex/Age (Years)	Underlying Condition(s)	Clinical Presentation	Infection Source	Identification Method	Other Co-Infected Pathogens	Antibiotic Treatment	Outcome
Agirbasli, H. et al., 2008 [7]	F, 8	AML	Respiratory distress	Lung	IGS1 sequencing	*Aspergillus fumigatus*	AMB (5 mg/kg/D) + IZ (100 mg D)	Death
Taj-Aldeen, S.J. et al., 2009 [10]	M, 31/M, 33	N.A.	Tinea pedis of the diabetic foot/Tinea pedis	Skin, intertrigo/Skin	ITS + 28S D1/D2 sequencing	N.A.	N.A.	N.A.
Bongomin, F. et al., 2019 [12]	F, 18	Severe hypotension and biventricular heart failure	Fungemia	Blood, aortic cannula, removed left ventricular apex cuff	ITS + 28S D1/D2 sequencing; MALDI-TOF MS	None	Prophylaxis FZ and CAS, AMB + 5FC, switch VOR	Survival at 2 months
Li, T. et al., 2020 [14]	M, 36/M, 50	Kidney transplant recipient	Urinary tract infection/Urinary tract infection	Urinary tract/Urinary tract	ITS sequencing; MALDI-TOF MS	*Candida glabrata*/None	VOR + CAS	Survival/Death
Albitar-Nehme S. et al., 2022 [8]	M, 8	ALL	Fungemia	Blood	MALDI-TOF MS; Molecular Typing ^a^	None	Prophylaxis AMB and CAS, AMB (3 mg/kg/D) + VOR (8 mg/kg twice a day)	Death
Menu, E. et al. 2022 [16]	F, 42	Congenital cardiopathy, severe left ventricular dysfunction (LVEF 25%) and New York Heart Association class III dyspnea.	Pericardial effusion, acute respiratory distress syndrome	Pericardial fluid, pericardial drain hole and the swab of the sternal surgery scar wound	MALDI-TOF MS; IGS1 sequencing	None	AMB (1.5 mg/kg/D)	Death
This study	M, 69	Diffuse large B-cell lymphoma, elevated blood glucose	Fungemia	Blood from central venous catheter and peripheral vein	ITS + IGS1 sequencing; MALDI-TOF	None	MF (2D), then AMB (2 mg/kg, 4 D), then AMB (4 mg/kg/D, 16 D) + VOR (4 mg/kg twice a day, 16 D)	Survival

Note: N.A. means Not Available, not mentioned in original article. Abbreviation: M male; F female; D Day. AML: Acute Myeloid Leukemia; AMB: Amphotericin B; IZ: Itraconazole; FZ: Fluconazole; CAS: Caspofungin; 5-FC: 5-fluorocytosine; VOR: Voriconazole; MF: micafungin. ^a^ Albitar-Nehme et al. reported using the MicroSEQ™ 500 16S rDNA PCR Kit for molecular typing, a method primarily designed for bacterial identification.

## Data Availability

All data generated or analyzed during this study are included in the article and Supplementary File. The ITS and IGS1 gene sequences have been deposited in the GenBank database under accession numbers PZ161416 (ITS) and PZ302131 (IGS1).

## Supplementary Materials

The following supporting information can be downloaded at: https://www.mdpi.com/article/10.3390/jof12050320/s1. File S1. FASTA format nucleotide sequences used for constructing the phylogenetic trees.

## Abbreviations

5-FC	5-fluorocytosine
AMB	amphotericin B
AND	anidulafungin
CAR-T	chimeric antigen receptor T-cell
CAS	caspofungin
CLSI	Clinical and Laboratory Standards Institute
CRP	C-reactive protein
DLBCL	diffuse large B-cell lymphoma
ECMM	European Confederation of Medical Mycology
EUCAST	European Committee on Antimicrobial Susceptibility Testing
FZ	fluconazole
G-CSF	granulocyte colony-stimulating factor
IGS1	intergenic spacer 1
IL-6	interleukin-6
ISHAM	International Society for Human and Animal Mycology
ITS	internal transcribed spacer
IZ	itraconazole
MALDI-TOF MS	matrix-assisted laser desorption/ionization time-of-flight mass spectrometry
MF	micafungin
MIC	minimal inhibitory concentration
PCT	procalcitonin
PZ	posaconazole
VOR	voriconazole
WBC	white blood cell
