# Supplementary material for: Catheter-Associated Trichosporon japonicum Fungemia in a Patient with Diffuse Large B-Cell Lymphoma Following CAR-T Cell Therapy: A Case Report and Literature Review
Source: J Fungi (Basel). 2026 Apr 27;12(5):320. doi: 10.3390/jof12050320 (PMC13208537; doi:10.3390/jof12050320)
Supplement: Supplementary file 1 [file jof-12-00320-s001.zip › Supplementary File 1.pdf]

>NR_073263.1 Trichosporon japonicum CBS 8641 ITS region; from TYPE material
TCCGTAGGTGAACCTGCGGAAGGATCATTAGTGATTGCCTTAATTGGCTTATAACTATATCCACT
TACACCTGTGAACTGTTCTACTACTTGACGCAAGTCGAGTATTTTTACAAACAATGTGTAATGAA
CGTCGTTTTATTATAACAAAATAAAACTTTCAACAACGGATCTCTTGGCTCTCGCATCGATGAAG
AACGCAGCGAATTGCGATAAGTAATGTGAATTGCAGAATTCAGTGAATCATCGAATCTTTGAAC
GCAGCTTGCGCTCTCTGGTATTCCGGAGAGCATGCCTGTTTCAGTGTCATGAAATCTCAACCACT
AGGGTTTCCTAATGGATTGGATTTGGGCGTCTGCGATCTCTGATCGCTCGCCTTAAAAGAGTTAG
CAAGTTTGACATTAATGTCTGGTGTAATAAGTTTCACTGGGTCCATTGTGTTGAAGCGTGCTTCTA
ATCGTCCGCAAGGACAATTACTTTGACTCTGGCCTGAAATCAGGTAGGACTACCCGCTGAACTT
AAGCATATCAATAAGCGGAGGA

>NR_155872.1 Trichosporon aquatile CBS 5973 ITS region; from TYPE material
TCCGTAGGTGAACCTGCGGAAGGATCATTAGTGATTGCCTTCATTGGCTTAAAACTATATCCACA
TACACCTGTGAACTGTTCTACTACTTGACGCAAGTCGAGTATTTTTACAAACAATGTGTAATGAA
CGTCGTTTTATTATAACAAAATAAAACTTTCAACAACGGATCTCTTGGCTCTCGCATCGATGAAG
AACGCAGCGAATTGCGATAAGTAATGTGAATTGCAGAATTCAGTGAATCATCGAATCTTTGAAC
GCAGCTTGCGCTCTCTGGTATTCCGGAGAGCATGCCTGTTTCAGTGTCATGAAATCTCAACCACT
AGGGTTTCCTAATGGATTGGATTTGGGCGTTGCGATCTCTGATCGCTCGCCTTAAAAGAGTTAGC
AAGTTTGACATTAATGTCTGGTGTAATAAGTTTCACTGGGTCCATTGTGTTGAAGCGTGCTTCTA
ATCGTCCGCAAGGACAATTACTTTGACTCTGGCCTGAAATCAGGTAGGACTACCCGCTGAACTT
AAGCATATCAATAAGCGGAGGA

>NR_073341.1 Trichosporon asahii CBS 2479 ITS region; from TYPE material
TCCGTAGGTGAACCTGCGGAAGGATCATTAGTGATTGCCTTTATAGGCTTATAACTATATCCACT
TACACCTGTGAACTGTTCTACTACTTGACGCAAGTCGAGTATTTTTACAAACAATGTGTAATGAA
CGTCGTTTTATTATAACAAAATAAAACTTTCAACAACGGATCTCTTGGCTCTCGCATCGATGAAG
AACGCAGCGAATTGCGATAAGTAATGTGAATTGCAGAATTCAGTGAATCATCGAATCTTTGAAC
GCAGCTTGCGCTCTCTGGTATTCCGGAGAGCATGCCTGTTTCAGTGTCATGAAATCTCAACCACT
AGGGTTTCCTAATGGATTGGATTTGGGCGTCTGCGATTTCTGATCGCTCGCCTTAAAAGAGTTAG
CAAGTTTGACATTAATGTCTGGTGTAATAAGTTTCACTGGGTCCATTGTGTTGAAGCGTGCTTCTA
ATCGTCCGCAAGGACAATTACTTTGACTCTGGCCTGAAATCAGGTAGGACTACCCGCTGAACTT
AAGCATATCAATAAGCGGAGGA

>NR_073241.1 Trichosporon asteroides CBS 2481 ITS region; from TYPE material
TCCGTAGGTGAACCTGCGGAAGGATCATTAGTGATTGCCTTAATTGGCTTATAACTATATCCACT
TACACCTGTGAACTGTTCTATTACTTGACGCAAGTCGAGTATTTTTACAAACAATGTGTAATGAA
CGTCGTTTTATTATAACAAAATAAAACTTTCAACAACGGATCTCTTGGCTCTCGCATCGATGAAG
AACGCAGCGAATTGCGATAAGTAATGTGAATTGCAGAATTCAGTGAATCATCGAATCTTTGAAC
GCAGCTTGCGCTCTCTGGTATTCCGGAGAGCATGCCTGTTTCAGTGTCATGAAATCTCAACCACT
AGGGTTTCCTAATGGATTGGATTTGGGCGTCTGCGATCTCTGATCGCTCGCCTTAAAAGAGTTAG
CAAGTTTGACATTAATGTCTGGTGTAATAAGTTTCACTGGGTCCATTGTGTTGAAGCGTGCTTCTA
ATCGTCCGCAAGGACAATTACTTTGACTCTGGCCTGAAATCAGGTAGGACTACCCGCTGAACTT
AAGCATATCAATAAGCGGAGGA

>NR_197550.1 Trichosporon austroamericanum CBS 17435 ITS region; from TYPE material
ACCTGCGGAAGGATCATTAGTGATTGCCTTTACAGGCTTAAACTATATCCACTTACACCTGTGAA
CTGTTCTACCACTTGACGCAAGTCGAGTGCTTTTACAAACAATGTGTAATGAACGTCGTTTTATT
ATAACAAAATAAAACTTTCAACAACGGATCTCTTGGCTCTCGCATCGATGAAGAACGCAGCGAA
TTGCGATAAGTAATGTGAATTGCAGAATTCAGTGAATCATCGAATCTTTGAACGCAGCTTGCGCT
CTCTGGTATTCCGGAGAGCATGCCTGTTTCAGTGTCATGAAATCTCAACCACTAGGGTTTCCTAA
TGGATTGGATTTGGGCGTCTTGCGATCTCTGATCGCTCGCCTTAAAAGAGTTAGCAAGTTTGACA
TTCATGTCTGGTGTAATAAGTTTCACTGGGTCCATGGTGTTGAAGCGTGCTTCTAATCGTCCGCA
AGGACAATTACTTTGACTCTGGCCTGAAATCAGGTAGGACTACCCGCTGAACTTAAGCATATCA
ATA

>NR_073333.1 Trichosporon caseorum CBS 9052 ITS region; from TYPE material
GTGATTGCCTTATAGGCTTAACTATATCCACATACACCTGTGAACTGTTCTACTACTTGACGCAA
GTCGAGTATTTTTACAAACAATGTGTAATGAACGTCGTTTTATTATAACAAAATAAAACTTTCAAC
AACGGATCTCTTGGCTCTCGCATCGATGAAGAACGCAGCGAATTGCGATAAGTAATGTGAATTG
CAGAATTCAGTGAATCATCGAATCTTTGAACGCAGCTTGCGCTCTCTGGTATTCCGGAGAGCAT
GCCTGTTTCAGTGTCATGAAATCTCAACCACTAGGGTTTCCTAATGGATTGGATTTGGGTGTTGC
GATCTCTGATCGCTCGCCTTAAAAGAGTTAGCAAGTTTGACATATATGTCTGGTGTAATAAGTTT
CACTGGGTCCATTGTGTTGAAGCGTGCTTCTAATCGTCCGCAAGGACAATTACTTTGACTC

>NR_073249.1 Trichosporon coremiiforme CBS 2482 ITS region; from TYPE material
TCCGTAGGTGAACCTGCGGAAGGATCATTAGTGATTGCCTTTATAGGCTTATAACTATATCCACT
TACACCTGTGAACTGTTCTATTACTTGACGCAAGTCGAGTATTTTTACAAACAATGTGTAATGAA
CGTCGTTTTATTATAACAAAATAAAACTTTCAACAACGGATCTCTTGGCTCTCGCATCGATGAAG
AACGCAGCGAATTGCGATAAGTAATGTGAATTGCAGAATTCAGTGAATCATCGAATCTTTGAAC
GCAGCTTGCGCTCTCTGGTATTCCGGAGAGCATGCCTGTTTCAGTGTCATGAAATCTCAACCACT
AGGGTTTCCTAATGGATTGGATTTGGGCGTCTGCGATCTCTGATCGCTCGCCTTAAAAGAGTTAG
CAAGTTTGACATTAATGTCTGGTGTAATAAGTTTCACTGGGTCCATTGTGTTGAAGCGTGCTTCTA
ATCGTCCGCAAGGACAATTACTTTGACTCTGGCCTGAAATCAGGTAGGACTACCCGCTGAACTT
AAGCATATCAATAAGCGGAGGA

>NR_073242.1 Trichosporon faecale CBS 4828 ITS region; from TYPE material
TCCGTAGGTGAACCTGCGGAAGGATCATTAGTGATTGCCTTTATAGGCTTATAACTATATCCACT
TACACCTGTGAACTGTTCTACTACTTGACGCAAGTCGAGTATTTTTACAAACAATGTGTAATGAA
CGTCGTTTTATTATAACAAAATAAAACTTTCAACAACGGATCTCTTGGCTCTCGCATCGATGAAG
AACGCAGCGAATTGCGATAAGTAATGTGAATTGCAGAATTCAGTGAATCATCGAATCTTTGAAC
GCAGCTTGCGCTCTCTGGTATTCCGGAGAGCATGCCTGTTTCAGTGTCATGAAATCTCAACCACT
AGGGTTTCCTAATGGATTGGATTTGGGCGTCTGCGATCTCTGATCGCTCGCCTTAAAAGAGTTAG
CAAGTTTGACATTAATGTCTGGTGTAATAAGTTTCACTGGGTCCATTGTGTTGAAGCGTGCTTCTA
ATCGTCCGCAAGGACAATTACTTTGACTCTGGCCTGAAATCAGGTAGGACTACCCGCTGAACTT
AAGCATATCAATAAGCGGAGGA

>NR_073243.1 Trichosporon inkin CBS 5585 ITS region; from TYPE material
TCCGTAGGTGAACCTGCGGAAGGATCATTAGTGATTGCCTTTACAGGCTTAACTATATCCACATA

CACCTGTGAACTGTTCTACCACTTGACGCAAGTCGAGTGTTTTTACAAACAATGTGTAATGAACG
TCGTTTTATTATAACAAAATAAAACTTTCAACAACGGATCTCTTGGCTCTCGCATCGATGAAGAA
CGCAGCGAATTGCGATAAGTAATGTGAATTGCAGAATTCAGTGAATCATCGAATCTTTGAACGC
AGCTTGCGCTCTCTGGTATTCCGGAGAGCATGCCTGTTTCAGTGTCATGAAATCTCAACCACTAG
GGTTTCCTAATGGATTGGATTTGGGCGTCTGCGATCTCTGATCGCTCGCCTTAAAAGAGTTAGCA
AGTTTGACATTCATGTCTGGTGTAATAAGTTTCACTGGGTCCATGGTGTTGAAGCGTGCTTCTAA
TCGTCCGCAAGGACAATTACTTTGACTCTGGCCTGAAATCAGGTAGGACTACCCGCTGAACTTA
AGCATATCAATAAGCGGAGGA

>NR_111353.1 Trichosporon insectorum ATCC MYA-4361 ITS region; from TYPE material
TTTCCGTAGGTGAACCTGCGGAAGGATCATTAGTGATTGCCTTTATAGGCTTATAACTATATCCA
CTTACACCTGTGAACTGTTCTACTACTTGACGCAAGTCGAGTATTTTTACAAACAATGTGTAATG
AACGTCGTTTTATTATAACAAAATAAAACTTTCAACAACGGATCTCTTGGCTCTCGCATCGATGA
AGAACGCAGCGAATTGCGATAAGTAATGTGAATTGCAGAATTCAGTGAATCATCGAATCTTTGA
ACGCAGCTTGCGCTCTCTGGTATTCCGGAGAGCATGCCTGTTTCAGTGTCATGAAATCTCAACC
ACTAGGGTTTCCTAATGGATTGGATTTGGGCGTCTGCGATCTCTGATCGCTCGCCTTAAAAGAGT
TAGCAAGTTTGACATTAATGTCTGGTGTAATAAGTTTCACTGGGTCCATTGTGTTGAAGCGTGCT
TCTAATCGTCCGCAAGGACAATTACTTTGACTCTGGCCTGAAATCAGGTAGGACTACCCGCTGA
ACTTAAGCATATCAATAAGCGGAGGA

>NR_073334.1 Trichosporon lactis CBS 9051 ITS region; from TYPE material
GTGATTGCCTTTATAGGCTTAACTATATCCACATACACCTGTGAACTGTTCTACTACTTGACGCA
AGTCGAGTATTTTTACAAACAATGTGTAATGAACGTCGTTTTATTATAACAAAATAAAACTTTCAA
CAACGGATCTCTTGGCTCTCGCATCGATGAAGAACGCAGCGAATTGCGATAAGTAATGTGAATT
GCAGAATTCAGTGAATCATCGAATCTTTGAACGCAGCTTGCGCTCTCTGGTATTCCGGAGAGCA
TGCCTGTTTCAGTGTCATGAAATCTCAACCACTAGGGTTTCCTAATGGATTGGATTTGGGTGTTG
CGATCTCTGATCGCTCGCCTTAAAAGAGTTAGCAAGTTTGACATATATGTCTGGTGTAATAAGTT
TCACTGGGTCCATTGTGTTGAAGCGTGCTTCTAATCGTCCGCAAGGACAATTACTTTGACTC

>NR_073254.1 Trichosporon ovoides CBS 7556 ITS region; from TYPE material
TCCGTAGGTGAACCTGCGGAAGGATCATTAGTGATTGCCTTCTAGGCTTAAACTATATCCACATA
CACCTGTGAACTGTTCTACCACTTGACGCAAGTCGAGTGTTTTTACAAACAATGTGTAATGAACG
TCGTTTTATTATAACAAAATAAAACTTTCAACAACGGATCTCTTGGCTCTCGCATCGATGAAGAA
CGCAGCGAATTGCGATAAGTAATGTGAATTGCAGAATTCAGTGAATCATCGAATCTTTGAACGC
AGCTTGCGCTCTCTGGTATTCCGGAGAGCATGCCTGTTTCAGTGTCATGAAATCTCAACCACTAG
GGTTTCCTAATGGATTGGATTTGGGCGTTGCGATCTCTGATCGCTCGCCTTAAAAGAGTTAGCAA
GTTTGACATTAATGTCTGGTGTAATAAGTTTCACTGGTTCCATTGTGTTGAAGCGTGCTTCTAATC
GTCCGCAAGGACAATTACTTTGACTCTGGCCTGAAATCAGGTAGGACTACCCGCTGAACTTAAG
CATATCAATAAGCGGAGGA

>ITS-MAOBAOZIJUN.19487099
CCTTCCGGTAGGGGGACCTGCGGAAGGATCATTAGTGATTGCCTTAATTGGCTTATAACTATATC
CACTTACACCTGTGAACTGTTCTACTACTTGACGCAAGTCGAGTATTTTTACAAACAATGTGTAA
TGAACGTCGTTTTATTATAACAAAATAAAACTTTCAACAACGGATCTCTTGGCTCTCGCATCGAT

GAAGAACGCAGCGAATTGCGATAAGTAATGTGAATTGCAGAATTCAGTGAATCATCGAATCTTT
GAACGCAGCTTGCGCTCTCTGGTATTCCGGAGAGCATGCCTGTTTCAGTGTCATGAAATCTCAA
CCACTAGGGTTTCCTAATGGATTGGATTTGGGCGTCTGCGATCTCTGATCGCTCGCCTTAAAAGA
GTTAGCAAGTTTGACATTAATGTCTGGTGTAATAAGTTTCACTGGGTCCATTGTGTTGAAGCGTG
CTTCTAATCGTCCGCAAGGACAATTACTTTGACTCTGGCCTGAAATCAGGTAGGACTACCCGCT
GAACTTAAGCATATCAATAAGGCGGGAGGAAA
